# Characterization of CMP Slurries Using Densitometry and Refractive Index Measurements

**DOI:** 10.3390/mi9110542

**Published:** 2018-10-24

**Authors:** Leticia Vazquez Bengochea, Yasa Sampurno, Marcus Kavaljer, Rob Johnston, Ara Philipossian

**Affiliations:** 1 Department of Chemical and Environmental Engineering, University of Arizona, Tucson, AZ 85721, USA; yasayap@email.arizona.edu (Y.S.); ara@email.arizona.edu (A.P.); 2 Araca Inc., Tucson, AZ 85718, USA; 3 K-Patents Oy, 01510 Vantaa, Finland; marcus.kavaljer@kpatents.com; 4 Yarbrough, a Watlow Company, Austin, TX 78758, USA; rjohnston@yswsemi.com

**Keywords:** semiconductor technology, chemical mechanical planarization, slurry characterization, in-line monitoring and control

## Abstract

We investigated the possibility of employing refractive index (RI) measurements for inline incoming slurry control at the point of use (POU), as an alternative to the widespread densitometry method. As such, it became necessary to determine if RI could detect smaller changes in slurry composition and, therefore, provide a tighter control. Three industrially-relevant silica-based slurries, namely, Fujimi PL-7106, Klebosol 1501-50, and CMC W7801, were characterized using both densitometry and RI measurements. Initial solutions of the three slurries were prepared and increasingly small amounts of ultrapurified water (UPW) were added to study the change in slurry properties. Results showed that both density and RI decreased linearly with the addition of water for all three slurries, with the 1501-50 being the most sensitive to water addition. A linear correlation between the two properties was found, with R^2^ values that exceeded 0.95 in all cases. Furthermore, the approximate limit of detection of both metrology tools was estimated based on the slope of the fitting line and resolution. When compared to densitometry, RI was found to be the far superior method for detecting smaller changes in water concentration.

## 1. Introduction

Chemical Mechanical Planarization (CMP) is a key enabling step in integrated circuits manufacturing. During this process, a wafer is pressed down against a polishing pad while both wafer and pad are rotated counterclockwise at slightly different velocities. As a slurry is dispensed on the center of the pad, the combination of mechanical and chemical actions results in material removal from the wafer surface, leading to a surface that is locally and globally planar. The slurry in CMP is a suspension of nanosized abrasive particles in an aqueous phase, which contains other chemicals such as oxidizers, organic acids, and complexing agents [1,2]. In recent years, the chemical compositions of slurries have become increasingly more complicated in order to satisfy the ever more stringent requirements imposed by the latest technology nodes. As a result, compounds such as surfactants, polyelectrolytes, pH adjusters, fungicides, bactericides, and heterocyclic organic compounds have been introduced into today’s advanced slurries. 

As minimum feature sizes have dropped below 10 nm, wafer-level planarity and defect specifications have become more stringent. This has caused CMP processes to become more complex and the demands on slurry quality control have become ever more stringent. Even though slurry control at the point of manufacture (POM) may be very tight, subsequent operations involving its transport, handling, blending, filtration and final dispense on the pad tend to cause changes in its chemical properties (i.e., in the oxidizer and additives concentrations). Such changes can affect process performance and increase wafer-level defects which will inevitably affect module productivity and yield. To prevent such undesirable consequences, chemical properties must be continuously monitored for the slurry at its point of use (POU) [3,4].

To this effect, rapid, reliable, precise, and cost-effective metrology tools and methodologies are required. Benchtop or offline methods, such as titration, have been widely used because they provide robust and accurate results. However, they have several limitations, including the inability to provide real-time information about the process, the possibility of introducing contamination during measurement, and a high operational cost. On the contrary, inline techniques provide continuous monitoring, thus allowing a much earlier detection of problems which would otherwise compromise wafer throughput. Although some accuracy is lost with inline methods, they tend to be more precise than offline techniques due to total automation [4,5,6].

Some of the slurry metrics that are currently being measured inline include density, conductivity, pH, absorption, and refractive index [7]. These techniques present advantages and disadvantages, and their selection depends on the specific application. Conductivity measurements are at times reliable and accurate, but the heterogeneous nature of today’s slurries, coupled with complex nanoparticle surface charge effects due to certain additives, places a tremendous strain on conductivity measurements. Therefore, small changes in the conductivity of the system may not entirely be due to compositional changes, but rather due to changes in the dynamic equilibrium that is present in the slurry. pH flowmeters are simple and cost-effective, but they cannot be used to simultaneously monitor changes in composition due to variabilities in the concentration of both water and hydrogen peroxide. As H_2_O_2_ is a non-ionic compound, it will not have an effect on the pH of the slurry. As such, this technique will not detect any change in hydrogen peroxide content. Moreover, nowadays, slurries are heavily buffered solutions, so the pH will change very little (or not at all) with the addition of water. This makes this technique unsuitable for precise slurry monitoring. IR and UV absorption methods are very useful for identifying the presence of compounds, but they cannot easily determine the concentrations of various species. Densitometry has become the standard metrology tool for incoming slurry monitoring. Fluctuations in density indicate a non-uniform slurry (i.e., higher concentration of large particles can occur at any given time) which can lead to changes in the removal rate and defects formation [4,5,7]. The most common instrument employed in industrial applications is the oscillation-type densitometer. Its sample cell is a U-tube, which is subjected to an electromagnetic field, and density is determined based on the cell’s fundamental vibration frequency. Although this method provides accurate results, its inline application is problematic, since most of the sample cell U-tubes have metallic parts that will come in contact with the slurry. Harsh chemicals coupled with abrasive nanoparticles present in CMP slurries can potentially corrode (due to electrochemical reactions) and gradually erode (due to persistent collisions between the inner parts of the cell and the nanoparticles within the slurry) the metallic parts of the sample cell, thereby causing contamination of the slurry within the factory’s slurry delivery system.

An alternative method, namely Refractive Index (RI) measurement, was first introduced in 2007 and used to monitor the concentration of added hydrogen peroxide to the slurry for metal CMP applications [7]. RI measurement is a simple, cost-effective, and accurate technique that provides real-time information on slurry composition. As slurries are water-based suspensions, their RI values should be similar to that of water (i.e., 1.33) but slightly different depending on the specific type and concentration of particles and chemicals present. As such, for a given slurry, a change in the hydrogen peroxide concentration can be correlated to a change in the RI [7,8]. However, for the successful implementation of a refractometer, precise calibration is needed for each type of slurry. RI varies nonlinearly with temperature and concentration, and this dependence is specific for each slurry type. Most published RI values are reported at a baseline temperature of 20 °C or 25 °C. As slurry temperature in the sub-fab may vary seasonally and from batch to batch, a precise calibration of the instrument is needed to compensate for these changes in temperature to accurately correlate any changes in RI with changes in slurry concentration [7]. For colloidal silica slurries, universal calibration values which give a precise compensation have been developed. 

The main goal of this study is to investigate the correlation between refractive index and density for three different colloidal silica CMP slurries currently used in high-volume manufacturing. Moreover, we plan to compare the approximate limit of detection of both methods to determine if RI can be used to detect smaller changes in the slurry composition as compared to densitometry.

## 2. Materials and Methods 

The three different slurries tested were the Fujimi PL-7106 copper slurry, the Klebosol 1501-50 slurry used for ILD CMP, and the CMC W7801 tungsten slurry. Publicly available information about their properties and the recommended mixing ratios at POU are shown in Table 1. In our tests, density and refractive index were measured after small amounts of ultrapurified water (UPW) were systematically added to the slurries to alter their concentration. The percent by volume of water added ranged from 0 to nearly 10%.

The metrology tools used were the Mettler Toledo Densito 30PX densitometer and the K-Patents Semicon Process Refractometer PR-33-S. Detailed schematics and descriptions of the tools can be found elsewhere [9,10]. The refractometer was installed inline within a custom-built flow loop which consisted of a 2-L slurry holding tank, a magnetically levitated pump (made by Levitronix), which caused the slurry to circulate through the loop, and a control station which allowed the user to set the speed of the pump. In our case, a flowrate of 6.5 L per min was found to empirically correlate to a pump speed of approximately 3200 RPM and used throughout all tests. RI data in the flow loop was logged to a data file using an ethernet connection to a laptop, at a frequency of 0.33 Hz. The resolution of the refractometer was 0.00001, where resolution is defined as the smallest change the display shows [11]. On the other hand, the Mettler Toledo densitometer was an offline instrument which provided discrete values of a given slurry’s density with a single value for every sample measured. This principle of operation of this instrument was based on the inherent oscillations of the U-tube within its sample cell (such a system was typical of most inline densitometers employed in manufacturing facilities). The resolution of the densitometer was 0.0001 g/cm^3^ [9].

An instrument repeatability test was first performed for the densitometer, as this is an offline instrument that provides discrete results, followed by a calibration test for the two metrology tools (both tests were done with the Fujimi PL-7106 slurry diluted with water, and without addition of hydrogen peroxide). For the densitometer repeatability test, one sample of slurry was manually measured 10 times resulting in 10 discrete values. After this test, calibrations were performed on six samples with increasing amounts of additional UPW. These samples were labeled from No. 1 to 6 where the concentrations of added water were adjusted to 0, 1.96, 3.85, 5.66, 7.41, and 9.09% *v*:*v*. For the offline densitometer calibration, two measurements were performed for each sample while for the refractometer calibration, approximately 1 L of Sample No. 1 (i.e., PL-7106 with no additional UPW) was introduced in the tank and circulated at approximately 6.5 L per minute for 20 min to ensure homogeneous fluid flow. The RI during the last minute of that period of time was recorded, and the average was reported. Next, UPW was added to the slurry in the tank to reach the concentration corresponding to Sample No. 2 and it was circulated for the same period of time, noting the average RI of the last minute. The same procedure was repeated to obtain the RI for the other samples.

Once the calibration step was completed, experiments with the three slurries were performed as follows. Approximately 2 L of initial suspensions were prepared for each slurry, adding UPW and hydrogen peroxide as per each manufacturer’s specification. The dilution ratios are presented in Table 1. It can be noted that hydrogen peroxide was added to PL-7106 and W7801, but not to 1501-50 as it was not called for. The experimental procedure was the same for each slurry and similar to the calibration test noted above. First, around 1.2 L of the initial suspension without any additional UPW was introduced in the flow loop (i.e., Sample No. 1) and circulated for 20 min. Data for RI was logged during the last 60 s with a sampling frequency of 0.33 Hz. Next, the flow loop was stopped, and 100 mL of the sample was withdrawn for offline densitometry. The flow loop was then filled up to 1.2 L, adding the necessary amounts of slurry and UPW to achieve the desired concentration for Sample No. 2. The fluid was circulated again for 20 min with RI data collection commencing during the last minute of the test. After stopping the flow loop and the withdrawal of another 100 mL of fluid for offline densitometry, the procedure was repeated for the other four samples. The density of every sample withdrawn from the flow loop was determined by performing five measurements per sample. A systematic temperature increase was observed in the samples circulated in the flow loop for all three slurries, resulting in a total increase from Sample No. 1 to Sample No. 6 of about 3 to 4 °C. This increase in temperature was attributed to the heat generated by the magnetically levitated pump. The temperature of the samples in the densitometer also increased accordingly, since they were drawn from the flow loop and measured immediately. As both density and refractive index are temperature dependent variables, all values need to be adjusted to a baseline temperature to allow a fair comparison. As such, temperature was recorded for every sample and the correction formulas shown in the next section were applied. As for the calibration values of the refractometer, the same universal values previously developed by the manufacturer for colloidal silica slurries were employed for all three cases.

## 3. Results

### 3.1. Repeatability and Calibration Tests

The first step in this study was to perform repeatability tests for the densitometer. Repeatability is defined as the variation caused by the instrumentation, or the variation observed when the same operator measures the same sample multiple times with the same instrumentation [11]. The result for all 10 density measurements for the Fujimi PL-7106 sample was identical (i.e., 1.0737 g/cm^3^) giving a standard deviation of zero. Although ambient conditions and the sample were the same, we expected there to be some variability in the measurements mostly due to factors such as ambient noise, human error or the nature of the instrument. The apparent lack of variability indicated that the densitometer was not sensitive enough to capture these possible sources of variability. As the refractometer was an inline instrument that reported a continuous measurement, we did not perform discrete repeatability test. Instead, the standard deviation of the values recorded during the last minute of every sample that was run was used as an indicator of repeatability. 

**Figure 1:**
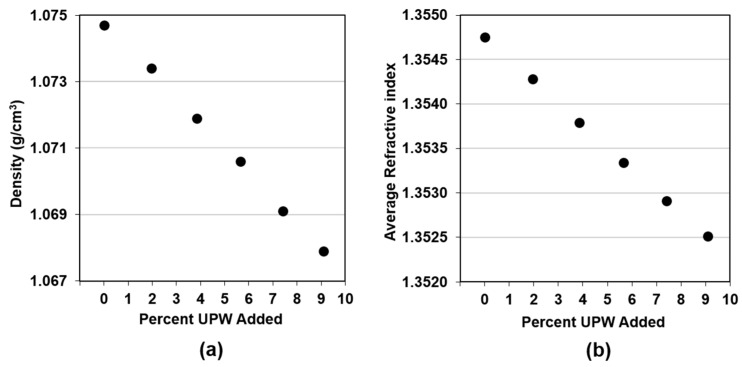
Calibration results as a function of amount of ultrapurified water (UPW) added for the Fujimi PL-7106 regarding (**a**) density and (**b**) average Refractive Index.

After the repeatability study, calibration tests were performed. Figure 1a shows the results for the density calibration test, where every point in the graph is the average of two measurements performed on each sample. Figure 1b shows the results for the RI calibration tests, with every point representing the average RI measured during the last minute of slurry circulation for every sample. It can be observed that both parameters decrease linearly with the addition of UPW. As shown in Table 1, the specific gravity of PL-7106 is higher than 1, so its density is expected to decrease with the addition of water. The decrease in RI indicates that PL-7106 is also optically denser than water.

### 3.2. Metrology

**Figure 2:**
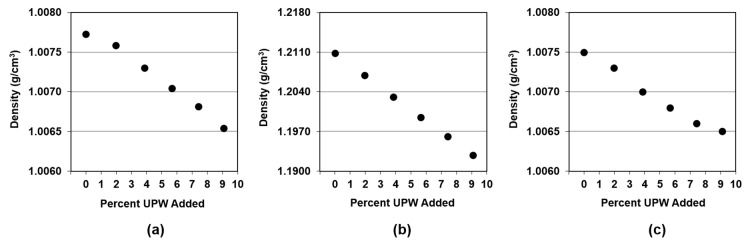
Slurry density as a function of amount of UPW added for (**a**) Fujimi PL-7106; (**b**) Klebosol 1501-50; and (**c**) CMC W7801.

Figure 2 shows density results for the three slurries as a function of added UPW. Every point represents the average of the five measurements performed for each sample. In all cases, the standard deviation of these five measurements is between zero and 9 × 10^−5^ g/cm^3^, as such, error bars are too small to be included in the figure. 

As density is a variable that depends on temperature, all the values have been adjusted to a baseline temperature of 25 °C as per Equation (1) [9].

d_c_ = d_m_∙[1 + α∙(T−T_o_)], (1)

where d_c_ is the corrected density in g/cm^3^, d_m_ is the measured density in g/cm^3^, T is the ambient temperature measured by the instrument in °C, T_o_ is the baseline temperature (25 °C), and α is the temperature compensation coefficient, which depends on the nature of the sample. As all slurries were water-based, the value for α corresponding to water (i.e., 0.0023 per °C) was used for all samples. We can see that in all cases, density decreased with the addition of water since the specific gravity of the three slurries was higher than unity (Table 1). A linear trend could be found for all slurries and we calculated the absolute value of the slope obtained by fitting a regression line to the experimental points. The slope was used as an approximation of the change in the output signal per unit change in the input signal [12]. The absolute values of the slopes for PL-7106, 1505-50, and W7801 were 0.000133, 0.00198, and 0.000115 g/cm^3^ per 1% additional UPW, respectively. As such, the 1505-50 proved to be most sensitive to changes in concentration and showed the highest change in density for the same amount of UPW added (i.e., one order of magnitude higher than PL-7106 and W7801). This was expected as the 1501-50 had the highest solids content (Table 1) which was affected the most by the addition of water. Consequently, one would expect that densitometry would be more suitable to monitor changes in 1501-50 than the other two slurries, because it could be able to detect smaller changes in its concentration. 

**Figure 3:**
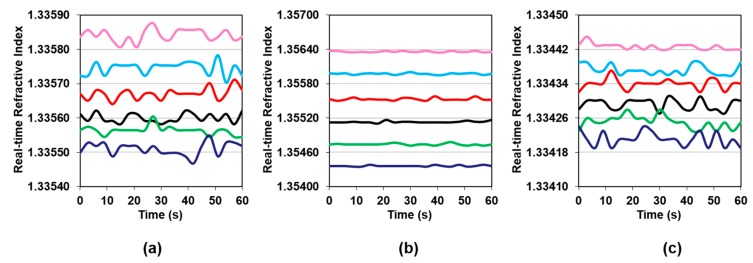
Inline refractive index vs. time for varying amounts of UPW added for (**a**) Fujimi PL-7106; (**b**) Klebosol 1501-50; and (**c**) CMC W7801. In all cases, Sample No. 1 is at the top, and No. 6 is at the bottom.

As explained earlier, RI measurements were recorded, and averaged, during a 60-s interval for every slurry after 20 min of circulation. Figure 3 shows the time trace of RI as logged using the K-Patents software for every sample of every slurry tested. Some fluctuations are observed in the signal during the 60-s interval. However, standard deviations for the six samples for each slurry ranged from 0.00001 to 0.00002. We must note that the Y-range for the graph corresponding to the Klebosol slurry is much larger than those for the other two slurries, yet the fluctuations in all three cases are of the same order, even if they look smaller in Figure 3b as compared to Figure 3a,c. This was close to the inherent resolution of the instrument which rendered our repeatability as being very good. 

**Figure 4:**
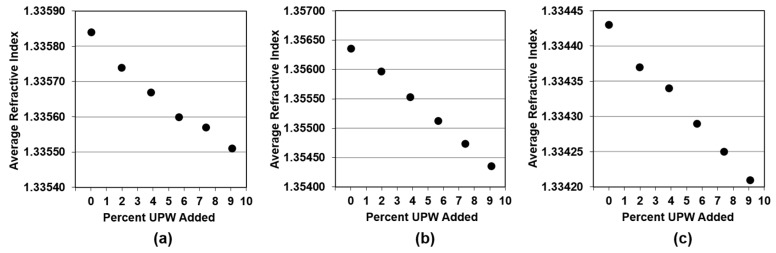
Average RI as a function of amount of UPW added for (**a**) Fujimi PL-7106; (**b**) Klebosol 1501-50; and (**c**) CMC W7801.

Figure 4 shows the average RI for every sample during the 60-s data-capture. As this property is also temperature dependent, the correction formula shown in Equation (2) (provided by the refractometer’s manufacturer) was applied to calculate the RI at a baseline temperature, which is selected to be the same as the density baseline (i.e., 25 °C).

RI_c_ = RI_m_∙[1 + β∙(T−T_o_)], (2)

where RI_c_ is the corrected refractive index, RI_m_ is the measured refractive index, and β is the temperature compensation coefficient, which depends on the nature of the sample. For all the experiments, we used the β value for water (i.e., 0.0001 per °C) as specified by K-Patents. It is apparent that RI decreases linearly with the addition of UPW for all the three slurries, since they are all optically denser than water. 

Similar to the densitometer, the change in output signal per unit change in input signal was approximated using the slope of the fitted line and reported to be 0.0000352, 0.000221, and 0.0000237 per 1% UPW for PL-7106, 1501-50, and W7801, respectively. As expected, 1501-50 seems to have the highest change in output, showing changes in RI that are one order of magnitude higher than PL-7106 and W7801 for the same change in UPW concentration. The 1501-50 slurry is also the optically densest (i.e., it has the highest values of RI among the three slurries) so it is highly affected by the addition of water. This trend is similar to the behavior of the three slurries regarding density, and we expect this tool to also be more suitable to monitor changes in concentration when applied to 1501-50 than the other two slurries.

When performing such dilution tests and measuring density and RI, one needs to question the issue that may arise regarding possible changes in the average diameter of the nanoparticle as a function of added water. After all, the colloidal silica particles in all three slurries are much denser and more optically opaque than water, and dilution may cause small amounts of particle stratification or agglomeration which will cause changes in the density or the opacity of the sample. In their study on colloidal silica particles, Nogowski et al. [13] found that large changes in dilution ratio (i.e., from 1:1 to 1:4 UPW:slurry) can affect the particle size distribution due to agglomeration. However, in our case, since small amounts of water are added, particle size distribution and, consequently, average particle size of the samples, are not expected to change. Indeed, using the 100 mL samples drawn from each of the diluted Sample Nos. 1 to 6, we proceeded to perform dynamic light scattering (using a Malvern Zetasizer Nano ZS instrument) to ensure that our tests were robust as they related to average particle size thus rendering credibility to our density and RI measurements. Tests confirmed the mean and standard deviation of the 6 samples for each slurry to be 68.3 ± 0.4, 58.5 ± 0.3, and 112.8 ± 0.7 nm for PL-7106, 1501-50, and W7801, respectively. The values for standard deviation were so low (ranging from 4 to 7 Angstroms) that we attributed them to the inherent measurement noise of the Zetasizer Nano ZS. We also checked the PSD for all samples in the three slurries, and we verified that they did not change with sample dilutions up to 9.09%.

### 3.3. Correlating Density with RI

**Figure 5:**
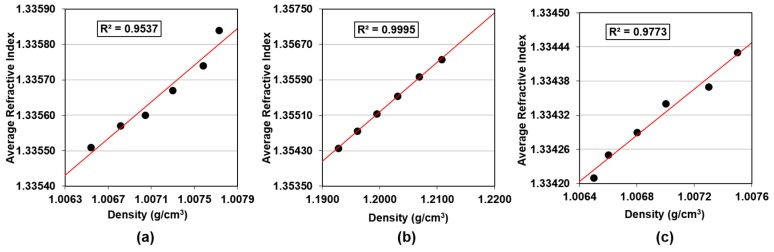
Correlation between density and average refractive index for (**a**) Fujimi PL-7106; (**b**) Klebosol 1501-50; and (**c**) CMC W7801.

As seen above, the three slurries show similar responses to UPW addition when it comes to their density and RI. Firstly, all three slurries show a linearly decreasing trend in both density and RI when water is added (see Figure 3 and Figure 4). Moreover, Klebosol 1501-50 is most sensitive to water addition as it experiences larger changes in RI and density for the same change in UPW concentration. Therefore, we believe that a correlation between these properties can be established as shown in Figure 5, where the three slurries clearly show a linear trend, with regression coefficients exceeding 0.95. 

From this information, the capability of both metrology tools for detecting changes in slurry concentration can be compared. The limit of detection (LOD) is defined as the lowest analyte concentration likely to be reliably distinguished from the blank sample, at which detection is feasible [14]. In our case, we would like to obtain and “Approximate LOD”, as the minimum change in concentration that could be detected by each instrument under ideal conditions, that is, without considering noise or experimental error. We estimate this limit to be represented as the ratio of the resolution of the instrument to the slope for each slurry. Table 2 shows the results for the “Approximate LOD” of densitometry and refractive index for the three slurries. The refractometer’s “Approximate LOD” is lower than that of the densitometer for all three cases. Therefore, the refractometer is able to detect smaller changes in slurry concentration. This is especially relevant for PL-7106 and W7801 slurries, which have much lower solids contents than 1501-50 (see Table 1). For these two slurries, the refractometer can resolve changes in UPW concentration that are 50% smaller than the minimum change detectable by the densitometer. It can also be observed that the Klebosol slurry, which contains a higher solids content, results in the highest regression coefficient for the correlation between density and RI, and the lowest LOD for both instruments. The high solids content leads to values of these properties that differ from water much more than for the other two slurries studied in this work. As such, any changes in water composition will have a more significant effect on both density and refractive index and will be easier to detect for the slurry that has a higher solids content. The refractometer has been proven to be a better option for the two slurries for which the solids content is low and the composition changes are more difficult to detect.

## 4. Discussion

This study investigated the feasibility of employing inline RI measurements as a means of monitoring and controlling incoming slurry composition. The method was compared and contrasted to the commonly employed method of densitometry. Three slurries containing silica abrasive particles were successfully characterized in this work. These included the Fujimi PL-7106 (for copper), the Klebosol 1501-50 (for ILD), and the CMC W7801 (for tungsten). Initial solutions of the three slurries were prepared as per manufacturers’ blending specifications, and increasing, small amounts of UPW were added to study the changes in density and RI. The results showed that both density and RI decreased linearly with the addition of water. This was expected since all three slurries have specific gravities and optical densities that were higher than that of water. 1501-50 was the most sensitive slurry regarding both properties, showing higher changes in both RI and density than PL-7106 and W7801 for the same change in water concentration. Dynamic Light Scattering was used to monitor the average particle size in all samples. The results indicated that no particle agglomeration occurred with the addition of UPW, so this particular metric was not affecting or biasing our measurements. Moreover, linear correlations between density and RI were found for the three slurries, with all regression coefficients exceeding 0.95. After the characterization step, the approximate limit of detection (LOD) of both methods was compared to determine as to which tool could detect smaller changes in water concentration and, therefore, be more suitable for tight incoming slurry monitoring. The refractometer was found to resolve smaller changes in UPW concentration for the three tested slurries. The difference was especially significant for PL-7106 and W7801, both containing lower solid content than 1501-50. 

## Figures and Tables

**Table 1:** Previously published properties and point-of-use blending specifications for each slurry.

Slurry	PL-7106	Klebosol 1501-50	CMC W7801
Manufacturer	Fujimi	Merck	Cabot Microelectronics
Application	Copper CMP	ILD CMP	Tungsten CMP
Nano-particle Type	Colloidal silica	Colloidal silica	Colloidal silica
pH	5.00–6.00	10.90	2.15–2.35
Nano-particle content (% by weight)	<1.5	30.0	<2.0
Specific Gravity	1.00–1.08	1.21	Not reported
Recommended POU Blending Ratios	UPW: PL-7106: H_2_O_2_(30%) 87.0:10.2:2.8	To be used as-received	W-7801: H_2_O_2_ (30%) 97.1:2.9

**Table 2:** Calculation of the approximate minimum detectable change in UPW concentration.

Slurry	Fujimi PL-7106		Klebosol 1501-50		CMC W7801	
Instrument	Densitometry	Refractive Index	Densitometry	Refractive Index	Densitometry	Refractive Index
Slope ($\frac{1}{\%\;UPW})$	1.33 × 10^−4^ $\frac{g}{{\mathrm{cm}}^{3}}$	3.52 × 10^−5^	1.98 × 10^−3^$\frac{g}{{\mathrm{cm}}^{3}}$	2.21 × 10^−4^	1.15 × 10^−4^ $\frac{g}{{\mathrm{cm}}^{3}}$	2.37 × 10^−5^
Approximate LOD (% UPW)	0.752	0.284	0.050	0.045	0.870	0.422
